# Car Transfer and Wheelchair Loading Techniques in Independent Drivers with Paraplegia

**DOI:** 10.3389/fbioe.2015.00139

**Published:** 2015-09-17

**Authors:** Lisa Lighthall Haubert, Sara J. Mulroy, Patricia E. Hatchett, Valerie J. Eberly, Somboon Maneekobkunwong, Joanne K. Gronley, Philip S. Requejo

**Affiliations:** ^1^Pathokinesiology Laboratory, Rancho Los Amigos National Rehabilitation Center, Downey, CA, USA; ^2^Rehabilitation Engineering, Rancho Los Amigos National Rehabilitation Center, Downey, CA, USA

**Keywords:** spinal cord injury, paraplegia, car transfer, shoulder pain, depression transfers, wheelchair, independent drivers

## Abstract

Car transfers and wheelchair (WC) loading are crucial for independent community participation in persons with complete paraplegia from spinal cord injury, but are complex, physically demanding, and known to provoke shoulder pain. This study aimed to describe techniques and factors influencing car transfer and WC loading for individuals with paraplegia driving their own vehicles and using their personal WCs. Sedans were the most common vehicle driven (59%). Just over half (52%) of drivers place their right leg only into the vehicle prior to transfer. Overall, the leading hand was most frequently placed on the driver’s seat (66%) prior to transfer and the trailing hand was most often place on the WC seat (48%). Vehicle height influenced leading hand placement but not leg placement such that drivers of higher profile vehicles were more likely to place their hand on the driver’s seat than those who drove sedans. Body lift time was negatively correlated with level of injury and age and positively correlated with vehicle height and shoulder abduction strength. Drivers who transferred with their leading hand on the steering wheel had significantly higher levels of shoulder pain than those who placed their hand on the driver’s seat or overhead. The majority of participants used both hands (62%) to load their WC frame, and overall, most loaded their frame into the back (62%) vs. the front seat. Sedan drivers were more likely to load their frame into the front seat than drivers of higher profile vehicles (53 vs. 17%). Average time to load the WC frame (10.7 s) was 20% of the total WC loading time and was not related to shoulder strength, frame weight, or demographic characteristics. Those who loaded their WC frame into the back seat had significantly weaker right shoulder internal rotators. Understanding car transfers and WC loading in independent drivers is crucial to prevent shoulder pain and injury and preserve community participation.

## Introduction

Shoulder pain is a common problem in individuals with spinal cord injury (SCI.) Its prevalence increases with time post-injury affecting as many as 70% of individuals by 20 years after SCI (Sie et al., 1992). The average age of onset of SCI is 41 years, and with advances in medicine, the life expectancy of individuals with SCI is approaching that of the non-disabled population^1^. Thus, persons with SCI have an extremely high likelihood of experiencing shoulder pain in their lifetime.

The development of shoulder pain in this population has been associated with the increase in upper limb weight-bearing demands for performance of transfers, pressure relief raises, and manual wheelchair (WC) propulsion. Shoulder pain negatively impacts independence and functional mobility, and is associated with significantly reduced subjective quality of life and physical activity in individuals with paraplegia (Gutierrez et al., 2007).

The demands of functional mobility on the shoulder joint following SCI have been explored in the laboratory, particularly during WC propulsion and depression raises and transfers. While depression transfers are performed less frequently than WC propulsion, median activity of key shoulder muscles is substantially greater, and glenohumeral contact forces are higher than that in WC propulsion. For instance, Perry et al. (1996) reported median pectoralis and infraspinatus major activity as high as 81 and 45% of maximum isometric contraction respectively, during the body lift of a level depression transfer in volunteers with low-level paraplegia vs. Mulroy et al. (2004) report median intensity of 34 and 20% during the push phase of self-selected free manual WC propulsion. Similarly, Van Drongelen et al. (2005) reported that the shoulder contact force was significantly greater (300%) during a weight relief lift than during level WC propulsion on an ergometer at 0.83 m/s.

Car transfers are the most demanding of transfers (Janssen et al., 1996), likely partly owing to the need to support the body across the gap between the WC and the car seat, resulting from the setback of the vehicle seat from the doorframe. Further, the vehicle seat is often higher than the WC seat, particularly for those transferring into higher profile vehicles, such as vans, SUVs, and trucks, increasing the demands on the trailing limb shoulder (Gagnon et al., 2008). Finally, for individuals driving independently, the need to load the WC into the vehicle presents an additional and substantial upper extremity demand. In spite of this increase in task demands, car transfer demands have not been documented in the literature.

Despite the high demands, car transfers are often a gateway to community mobility. Independent driving is the key to vocational engagement and community participation in individuals with SCI. Independent drivers with SCI were almost twice as likely to be engaged in a vocation (paid or volunteer work or school) as non-drivers (Hatchett et al., 2009) and demonstrated higher community reintegration and health-related quality of life scores compared to non-drivers (Norweg et al., 2011). Independent driving for persons who are dependent on a WC for mobility requires either car transfers or a modified van with a lift. Thus, the importance of understanding shoulder demands during car transfer and WC loading in order to prevent shoulder pain and injury and preserve independence and community participation for individuals with spinal cord injury is crucial.

The purpose of this study was to delineate the factors influencing car transfer and WC loading demands on the shoulders of individuals with paraplegia from SCI. We additionally endeavored to document transfer and WC loading times and techniques during transfer from individuals own WCs to their own vehicles.

## Materials and Methods

Volunteers with paraplegia resulting from SCI [American Spinal Injury Association Impairment Scale (AIS) A, B, or C] were recruited from outpatient clinics at Rancho Los Amigos National Rehabilitation Center (RLANRC) (a sample of convenience). Participants signed an informed consent approved by the Rancho Los Amigos Institutional Review Board prior to collection of observational data in the Pathokinesiology Laboratory and the parking lot at RLANRC. Individuals were at least 18 years of age with a minimum duration of paraplegia from SCI (AIS A, B, or C) of 2 years and maximum duration of 21 years. Volunteers were included if they pushed a manual WC for at least 50% of their locomotion and independently drove a vehicle and loaded their WC into their vehicle, according to self-report. Participants were excluded if they had cervical radiculopathy, adhesive capsulitis, a positive Codman’s Drop Arm Test or a history of shoulder or upper limb traumatic injury, fracture or surgery impacting function at the time of consent. They were additionally excluded if they had co-morbidities that could affect the integrity of their musculoskeletal system (e.g., rheumatoid arthritis, diabetes mellitus) and/or their body weight exceeded 250 lbs.

After reviewing and signing an informed consent in accordance with the Declaration of Helsinki, demographic data (personal factors), physical measurements, and WC measurements were collected from each participant. Vehicle measurements were taken prior to videotaping of the car transfer and WC loading. Demographic data included gender, age, level of and duration of SCI, and completeness of SCI. Physical measurements included body weight and height. WC measurements included weight of the WC frame and wheels, seat width, and seat height (from ground to the top of the WC cushion). Vehicle measurements included driver’s seat height (from the ground), roof height (from the ground to the top of driver’s door opening), and maximum driver’s side door opening. Additionally, the distance of the gap between the right side of the WC seat and the left side of the driver’s seat was measured once the volunteer had positioned their WC just prior to transferring into the vehicle. Vehicles were classified as one of the two heights: (1) low profile (sedans) and (2) high profile. The higher profile group consisted of (a) mid-height vehicles [vans and small to medium sports utility vehicles (SUVs)] and (b) high-profile vehicles (large trucks and SUVs), and were collapsed into one higher profile group for statistically meaningful results since only four volunteers drove high-profile vehicles (large trucks and SUVs). A Wheelchair User’s Shoulder Pain Index (WUSPI) form was completed prior to bilateral shoulder strength testing.

Bilateral maximal isometric shoulder torques were measured in six directions (abduction, adduction, flexion, extension, external rotation, and internal rotation) using a Biodex System 3 Pro Dynamometer (Biodex Medical Systems, Inc., New York, NY, USA). Volunteers were tested in a seated position with the trunk and pelvis secured by two chest straps and a pelvic strap. Shoulder abduction and adduction were tested at 45° of shoulder abduction, the elbow and wrist extended to neutral, and the forearm pronated 90°. Shoulder flexion and extension were tested with the shoulder flexed 45° and the elbow, wrist, and forearm neutral. External and internal rotation were tested with the UE positioned in 90° of abduction, the elbow in 90° of flexion, the wrist neutral, and the forearm pronated. Each participant was instructed to push or pull against the lever arm using their maximum effort for a duration of 5 s. Following one 3-s practice repetition to familiarize the participant with the test, two trials were performed with a 10–15 s rest break between trials. The peak values for each of the two trials for each UE were averaged and then normalized to body weight.

Participants were videotaped while transferring into and out of their own vehicle and loading their personal WC into and out of the vehicle. Two to three video cameras were utilized to capture the functional tasks from different angles [driver’s side sagittal view, passenger’s side sagittal view, and driver’s side diagonal (45–60° anterior to the sagittal plane)]. The driver’s side and passenger doors and windows were open to allow adequate video capture of the tasks without obstruction by the vehicle. Video was captured at a rate of 30 frames/s and was initiated as the subject approached the vehicle in the WC just prior to stopping next to driver’s seat of the vehicle to initiate the car transfer. Video recording was ended just after the volunteer loaded the last WC component in the vehicle and placed the hands on steering wheel, indicating completion of the loading task. Participants repeated the transfer and loading two times if they were able.

Videotape of each volunteer performing a transfer from their personal WC into the driver’s seat of their usual vehicle as well as loading of their WC, utilizing their customary technique, into the vehicle were reviewed by one physical therapist with 15 years of experience. All participants removed and loaded the wheels and many removed the cushion and/or sideguards from the WC frame prior to loading the frame into the front or back seat of the their vehicle. Movement strategies and transfer and loading times were documented. During the transfer into the vehicle, placement of the lower limbs prior to transfer was documented, location of the right and left hand just prior to and during the transfer, and number of scoots prior to the body lift onto the driver’s seat and afterward (just prior to WC loading) were recorded. Total transfer time (just after the WC is positioned to just prior to initiation of the reach for the WC for loading) and body lift time (from initiation of trunk lift to cessation of trunk descent) were quantified from time-stamped video. During WC loading, location of final resting place of the WC frame and components after loading into the vehicle was documented. Total loading time (from the initiation of reach for the WC to the placement of the last WC component in the vehicle and return to the driving position) and WC frame (heaviest component) loading time were quantified.

### Statistics

Data were analyzed using SPSS 12.0 Software (SPSS, Inc., Chicago, IL, USA). Bivariate Pearson product-moment correlations (*r*) were calculated between body lift time and participant demographics including age, level of SCI, body weight, maximal isometric torques of the shoulder muscle groups, and characteristics of the WC and car including car seat height, WC to car height difference, and horizontal distance between WC seat and car (gap). Correlations were also calculated between WC frame loading time and participant demographics, isometric shoulder torques, and WC frame weight. Chi-square tests were used to assess associations between vehicle heights (low vs. high profile) and hand and leg placement strategies during transfer and WC frame placement during loading. One-way analyses of variance (ANOVAs) tests were conducted to compare isometric muscle torques of the shoulder muscles and WUSPI (shoulder pain) scores between participants who used the various patterns of hand placement for right and left hands and leg placement during the body lift phase of the transfer. If a significant main effect of hand or leg placement strategy was found, a Tukey’s *post hoc* test was used to determine significance in pair-wise comparisons. An independent *t*-test was used to compare isometric muscle torques of the shoulder muscles and WUSPI (shoulder pain) scores between participants who loaded their WC frame in the front seat with those who placed it on the back seat. A *p*-value of 0.05 was used to denote statistical significance.

## Results

Participants included four females and 25 males with T2 to L3 (2–15) paraplegia (mean = 9.1 ± 3.5, Table 1) and an average age of 40.2 ± 8.8 years and average time since injury of 14.9 ± 7.9 years. Average body weight was 73.2 kg. Average driver’s seat height measured from the ground for the low-profile vehicles was 22 in, 28 in for the mid-height vehicles, and 36 in for the high profile. Most participants drove sedans (17 out of 29), seven drove mid-height, and five drove high profile vehicles. The average height difference between the WC seat and vehicle driver’s seat was 3.7 ± 5.5 in and ranged from −3.5 to 16.0 in.

**Table 1:** **Pearson product-moment correlations between body lift time and demographic, car/WC dimensions, and isometric shoulder torques**.

Variable	Mean (SD)	Range (min–max)	Pearson correlation (*r*) with body lift time	*p*-Value of correlation
Age (years)	40.2 (8.8)	22.9–61.1	−0.294	0.061
Duration of SCI (years)	14.9 (7.9)	3.1–35.5	−0.002	0.500
Level of SCI (T2 = 2, T3 = 3, …, T12 = 12, L1 = 13, L2 = 14, L3 = 15)	9.1 (3.5)	2–15 (T2–L3)	−**0.490**	**0.004**
Body weight (kg)	73.2 (17.1)	44.0–120.3	−0.203	0.145
Car seat height (in)	25.8 (5.3)	19.0–38.3	**0.426**	**0.011**
WC seat to car seat height difference (in)	3.7 (5.5)	−3.5 to 16.0	**0.408**	**0.014**
Horizontal distance between WC seat and car seat (in)	11.8 (2.2)	7.0–16.8	0.015	0.471
Number of scoots prior to body lift	1.32 (0.91)	0–5	−0.086	0.331
Right adduction (Nm/kg × 100)	98.4 (32.7)	78.6–91.3	0.173	0.184
Right internal rotation (Nm/kg × 100)	46.3 (18.7)	42.4–50.4	0.015	0.468
Right external rotation (Nm/kg × 100)	46.5 (14.4)	39.2–45.6	0.298	0.058
Right flexion (Nm/kg × 100)	89.5 (28.4)	71.1–81.4	0.240	0.105
Right abduction (Nm/kg × 100)	73.7 (25.3)	59.9–69.6	**0.337**	**0.037**
Right extension (Nm/kg × 100)	94.6 (31.0)	77.2–90.6	0.165	0.196
Left adduction (Nm/kg × 100)	99.9 (34.0)	78.6–91.3	0.134	0.244
Left internal rotation (Nm/kg × 100)	50.9 (16.8)	42.4–50.4	0.083	0.334
Left external rotation (Nm/kg × 100)	48.1 (14.6)	39.2–45.6	0.257	0.089
Left flexion (Nm/kg × 100)	90.7 (26.1)	71.1–81.4	0.162	0.201
Left abduction (Nm/kg × 100)	75.8 (24.1)	59.9–69.6	**0.349**	**0.032**
Left extension (Nm/kg × 100)	89.5 (27.1)	77.2–90.6	0.100	0.302

*Bold results indicate *p* < 0.05*.

### Description of transfer strategies

**Figure 1:**
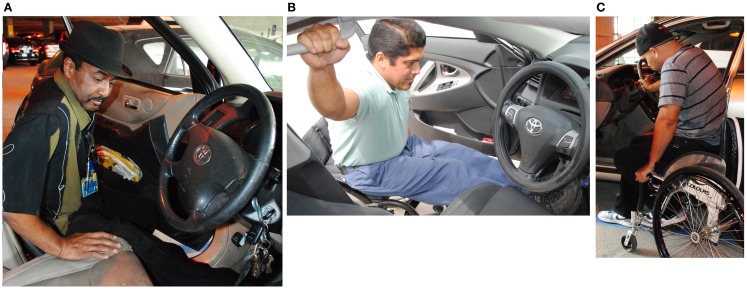
**(A)** Car transfer into a mid-height vehicle with right leg inside and left leg outside of the vehicle and leading hand on the driver’s seat. **(B)** Car transfer into a sedan with both legs in the vehicle and the leading hand on the grab bar. **(C)** Car transfer into a sedan with both legs outside of the vehicle and the leading hand on the steering wheel.

The majority of participants (15 out of 29) placed their right leg only into the car prior to the body lift phase of the transfer; five placed both legs in the car prior to the transfer, and eight transferred with their legs outside of the car (Figures 1A–C). The right or leading hand was most frequently placed on the driver’s seat (19/29) during the body lift portion of the transfer, while seven transferred with the right hand on the steering wheel and three transferred with the right hand on the overhead door frame or grab bar (Figures 1A–C). The left hand or trailing hand was placed most frequently on the WC seat for the body lift (14/29), while three placed their left hand on the steering wheel of the car, two on the wheel of the WC, three on both the WC seat and wheel, five on the driver’s side door, and two on the overhead door frame.

Vehicle height tended to influence hand placement but not leg placement strategy used during the body lift phase of car transfer. Drivers of higher profile vehicles were more likely to place their right hand on the driver’s seat during the body lift than those who drove sedans (10/12 mid and high profile vs. 9/17 sedan) (Chi square = 2.88, *p* = 0.09) In contrast, those who drove a sedan were more likely to place their right hand on other locations [steering wheel (5/17) or overhead door frame (3/17)] than higher profile vehicle drivers (steering wheel 2/12). Sedan drivers were somewhat more likely to place their left hand on the WC seat or wheel than those who drove higher profile vehicles (13/17 vs. 6/12). A minority of sedan driver’s (4/17) placed their left hand on various parts of the car (steering wheel = 2/17, overhead door frame = 1/17, and driver’s door = 1/17). In contrast, half of the drivers of higher profile vehicles (6/12) placed their left hand on the vehicle: steering wheel = 1/12, overhead door frame = 1/12, and driver’s door = 4/12. The association between car height and left hand placement did not reach statistical significance (*p* = 0.14). There was no association between car height and leg placement strategy.

### Body lift time

**Figure 2:**
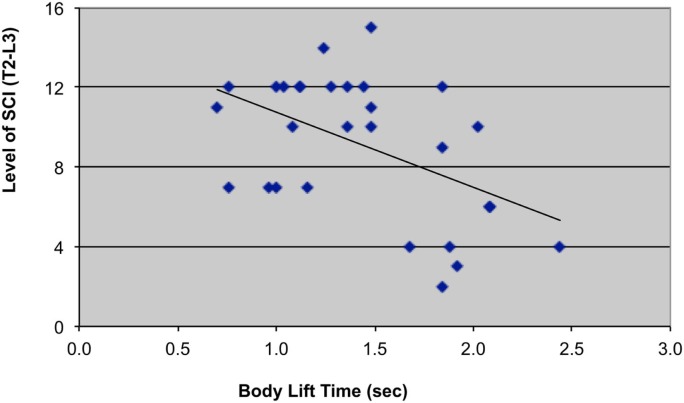
**Scatterplot with trend-line illustrating relationship between level of SCI (T2–L3) and body lift time (sec *=* seconds)**. T2 = 2, T3 = 3, T4 = 4, …, T12 = 12, Ll = 13, L2 = 14, L3 = 15.

**Figure 3:**
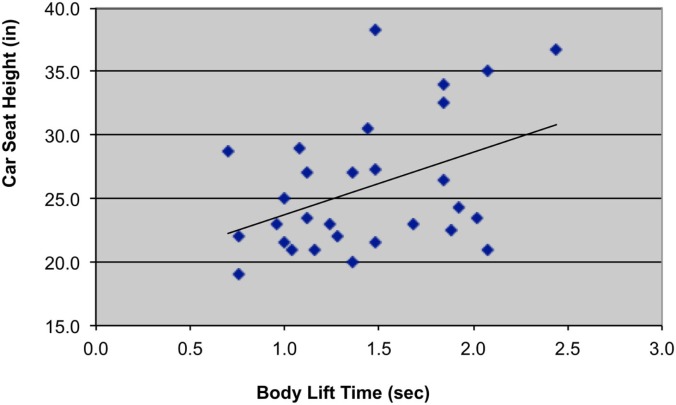
**Scatterplot with trend-line illustrating relationship between car seat height (in *=* inches) and body lift time (sec *=* seconds)**.

Mean body lift time was 1.43 ± 0.5 s, <10% of total transfer time (mean = 17.8 ± 10.5 s). Body lift times were negatively correlated with levels of paraplegia (*r* = −0.49, *p* = 0.004) (Table 1; Figure 2) and positively correlated with car seat height (*r* = 0.43, *p* = 0.011) (Figure 3). In addition, right and left shoulder abduction strength was positively correlated with body lift time (*r* = 0.34, *p* = 0.037 and *r* = 0.35, *p* = 0.032, respectively) and age was negatively correlated (*r* = −0.29, *p* = 0.061). Younger and stronger persons had longer body lift times than older and weaker individuals.

### Hand placement and shoulder pain

Participants who placed their right hand on the steering wheel during transfer had higher WUSPI scores indicating more pain (9.9 ± 15.7), compared to those who placed their right hand on the driver’s seat (1.3 ± 2.6) or overhead door frame (0.8 ± 1.3) (*p* = 0.05).

### Description of WC loading strategies

Most participants used both hands to load the WC frame (18/29), seven used only one hand and four lifted part of the time with one hand and part of the time with both hands. Just under half of participants did not rest the frame momentarily on the car or their legs during loading (13/29) and more loaded their WC frame onto the back seat (18/29) than onto the front seat (11/29). Of those who drove a sedan, 53% (9/17) placed their WC frame on the front seat, while only 17% (2/12) who drove higher profile vehicles placed the frame in the front seat (Chi-square = 3.38, *p* = 0.066).

### WC frame loading time

The average time to load the WC frame into the vehicle was 10.7 ± 7.9 s, which was 20% of the total WC loading time of 49.5 ± 21.8 s. Mean weight of the WC frame was 7.7 ± 2.1 kg. WC frame loading time was not related to isometric shoulder torques, weight of the frame, or any demographic characteristic.

### WC frame placement during loading

Participants who customarily placed their WCs in the back seat had weaker muscle strength in the internal rotators of the right arm (28.1 ± 9.3 Nm/kg × 100) compared to those who placed their WCs in the front seat (40.2 ± 10.3 Nm/kg × 100) (*p* = 0.003).

## Discussion

This study provides a descriptive analysis of customary movement strategies used by independent drivers with paraplegia from SCI to transfer from their WC into the driver’s seat and to load their WC frame into their vehicle. The most physically demanding portion of a car transfer is the sub-phase when the body weight is lifted off of the WC seat with the arms and to a lesser extent, the legs and placed into the car seat. The duration of this high-intensity phase (body lift) was related both to the individual’s level of SCI and to the height of the vehicle (Figures 2 and 3). Persons with lower levels of paraplegia spent less time in the body-lift phase of the transfer than those with higher paraplegia (Figure 2). A higher level of paraplegia results in a greater loss of innervation and strength in trunk muscles and has been associated with increased trunk flexion during “standard sitting pivot transfers” (depression transfers between two level, adjacent surfaces) (Desroches et al., 2013b). This increased trunk flexion was thought to increase stability by lowering the center of mass of the trunk and increasing the base of support. It also increased the average linear displacement of the body’s center of mass (Desroches et al., 2013a), which could result in a longer duration of the body lift phase. The body lift phase of the transfer also was longer when transferring into cars with higher driver’s seat heights, reflecting an increased demand on the shoulder with higher profile vehicles. Likely related was the association of younger age and stronger shoulder abductors with longer body lift times as younger and stronger persons tended to drive the higher profile vehicles.

The transfer times documented in the current study were similar but slightly longer than times reported in the literature for sitting pivot transfers. Sitting pivot transfers have been explored biomechanically in the laboratory by various groups (Allison et al., 1995, 1996; Perry et al., 1996; Allison, 1997; Allison and Singer, 1997; Nawoczenski et al., 2003; Gagnon et al., 2009; Desroches et al., 2012) and investigators differed in methodologies used to define the phases of transfer. For example, Nawoczenski et al. (2003) utilized angular and linear motion of the thorax to determine a preparatory, lift/pivot, and sit-back phase, while Gagnon et al. (2009) utilized a combination of kinematic and kinetic data to determine the pre-lift, lift, and post-lift phase of sitting pivot transfers. Additionally, sitting pivot transfers evaluated in the laboratory have consisted of transfers between levels, adjacent surfaces, unlike the typical car transfers assessed in the current study. Our goal was to define and quantify the most demanding portion of the car transfer, the body lift, which largely occurred from the lower WC surface to the driver’s seat that was additionally separated by a horizontal gap of 9–17 in, which is substantially larger than the separation of surfaces in most studies of sitting pivot transfers.

In spite of the higher demands of the car transfer, the average duration of the body lift phase of the car transfer reported in this study was largely consistent with sitting pivot transfer times documented in the literature. While definitions and procedures for determination of phasing varied between studies, Desroches et al. (2012) found that the lift-pivot phase, the most comparable to the body-lift phase quantified in our study, averaged 0.72 ± 0.24 s (vs. the average body lift duration of 0.915 s (0.515–1.072 s) into the sedan in the current study. It follows that a car transfer, a more complex and demanding version of the sitting pivot transfer, would take longer, particularly in higher profile vehicles when both the horizontal and vertical distances traversed were greater than in sitting pivot transfers.

Leading hand placement was associated with shoulder pain during transfer into the driver’s seat. Individuals who placed their leading hand on the steering wheel (24%) had significantly higher WUSPI scores than those who placed their hand on the driver’s seat (66%) or overhead on the grab bar or doorframe (10%). This might be explained by the degree of elevation of the weight-bearing shoulder with the leading hand on the steering wheel, which is most consistent with average angles documented by Bey et al. (2007). Bey and colleagues utilized biplane X-rays and subject-specific CT models to determine that the supraspinatus tendon is in closest proximity to the acromion between 27.7° and 36.1° of elevation. These findings suggest that impingement of the subacromial structures might be more likely with the leading hand on the steering wheel, particularly with repetition of this high-demand task.

Strategies for hand placement, but not leg placement, were influenced by vehicle height. Eighty-three percent of those driving higher profile vehicles placed their leading hand on the driver’s seat, while only 53% of sedan driver’s utilized this technique. Given the larger discrepancy between the WC and driver’s seat heights with higher profile vehicles, the leading hand may not be able to sufficiently reach the steering wheel or overhead grab bar or doorframe for theses transfers, except in taller individuals. Further, sedan drivers were more likely to place their trailing hand on the WC seat or wheel (77%) compared to higher profile vehicle drivers (50%). Individuals who routinely placed their WC frame onto the back seat of the car had isolated weakness in the internal rotator muscles of the right shoulder compared to those who placed their WC frame onto the front seat. Since the typical motion to place the frame into the back seat involves extreme external rotation with horizontal abduction of the shoulder, the internal rotators would need to control the weight of the frame during release. The typical shoulder position during release of the chair also happens to be the position to test for anterior instability of the shoulder. Gold et al. (2007) created a computer-generated model of the rotator cuff tendons from an open MRI during this test. They demonstrated that as the arm moves from a resting position at the side of the body to the extreme position of abduction and external rotation, the tendon of subscapularis, a primary internal rotator, is stretched significantly and lies directly beneath the acromion. Our participants are holding a WC frame that averages 7.7 kg so that the weakness of the internal rotation seen in those who put their chair in the back seat may well represent repetitive stretch-induced muscle injury.

### Limitations

Determination of transfer phasing was conducted by observation of events from a videotape. Given that our goal was to explore and describe car transfers in the real-world, we did not record kinematic and kinetic data for more objective determination of transfer times. However, video tape of the transfer from two to three different angles was utilized by a single physical therapist with 15 years of experience for all subjects to maximize consistency and accuracy.

Our sample size of 29 participants was low, given the variation in car transfer strategies and vehicle heights and relatively few participants had shoulder pain. Consequently, the results should be viewed as a preliminary study. Additionally, since only four volunteers drove high profile vehicles (large trucks and SUVs), it was necessary to collapse the medium and high profile vehicle groups into one group for statistically meaningful results.

## Conclusion

Documentation of the techniques utilized for car transfer from different WCs into a variety of vehicles and the loading of these WCs is a necessary preliminary step to optimize future biomechanical testing in the laboratory. We found that individuals with higher levels of injury, stronger shoulders, and higher profile vehicles had longer body lift times, relating to more demanding car transfers. Technique, particularly placing the leading hand on the steering wheel, was also associated with higher levels of shoulder pain. Placement of the WC frame, the heaviest component of the WC, into the back seat was associated with weakness in the internal rotators of the right shoulder, which may indicate injury from repetitive high demands on lengthened muscles. These findings illustrate the need to incorporate strength training into the weekly routines of individuals with SCI, a disability that places increased demands on the upper extremities. The ultimate goal is to determine optimal car transfer techniques to prevent shoulder pain and injury and maximize functional independence and participation for individuals aging with spinal cord injury.

## Conflict of Interest Statement

The authors declare that the research was conducted in the absence of any commercial or financial relationships that could be construed as a potential conflict of interest.

## Funding

Funded by NIDRR grant number H133E080024.

## Footnotes

^1^https://www.nscisc.uab.edu/, SCI Facts and Figures at a Glance, February 2012.
